# Novel in-home COVID-19 vaccination program for vulnerable populations using public-private collaboration

**DOI:** 10.3389/fpubh.2022.898787

**Published:** 2022-09-23

**Authors:** Megan S. Zhou, Cyrus Attia, Melynda Barnes, Tina Chen, Katie Chlada, Mel Doukas, Julia John, Julia Kanter, Dayna Kim, Kerry Qualliotine, Jillian Stein, Kevin Stern, Lauren Broffman

**Affiliations:** Ro, New York, NY, United States

**Keywords:** COVID-19, vaccine, older adults, vulnerable populations, in-home care

## Abstract

**Background:**

The continued emergence of new COVID-19 variants highlights the importance of vaccination in the effort to reduce disease transmission and burden. The objective of this study is to evaluate the processes and outcomes associated with a novel in-home COVID-19 vaccination program aimed at vaccinating high-risk populations in New York, USA.

**Methods:**

To evaluate program processes, we described the program itself and reflected on some key lessons learned. To evaluate program outcomes, we analyzed data reported by vaccine recipients. These outcomes included the percentage of vaccine recipients that successfully received the full course of vaccinations, and the demographic and health characteristics of vaccine recipients. We additionally assessed demographic differences in motivations for receiving in-home care, using chi-squared tests to assess statistical significance. Data were collected and reported via dynamic online intake forms.

**Results:**

The median age of vaccine recipients was 79 ± *SD* 9.0 years. The oldest vaccine recipient was 107 years old. Of those with non-missing data, more than half of vaccine recipients were female (63%), identified as part of a racial/ethnic minority (66%), reported an annual income of < $25,000 (58%), and received a high school degree or less (68%). Most vaccine recipients reported having one or more health conditions associated with increased risk of severe COVID-19 disease (72%). Vaccine recipients were most likely to report receiving in-home vaccination because they were home-bound due to disability. Motivations for receiving in-home vaccination differed by demographic subgroup.

**Conclusion:**

The population receiving vaccinations from this in-home care delivery program comprised seniors who were mostly female and non-white, indicated socioeconomic vulnerability, and reported one or more COVID-related health conditions; this signified that the program met its goal of vaccinating those most at risk for severe COVID-19 disease.

## Introduction

The continued emergence of new COVID-19 variants such as Omicron (B.1.1.529) and Delta (B.1.617.2) highlights the importance of vaccination as the best public health measure to reduce disease transmission and burden (1). To mitigate the especially high risk of severe COVID-19 disease in US adults 65 years and older, the Centers for Disease Control urgently recommend vaccination and boosters, which have been 95% effective in reducing hospitalization in the elderly population (2). However, individuals in this population are also more likely to face age-related vaccination barriers. Digital access barriers can both limit their access to healthcare resources and hinder their ability to register for a vaccine appointment (3), which can be challenging regardless of age (4). In addition to digital access barriers, seniors are also more likely to have health conditions that increase risk of severe COVID-19 disease (5, 6). Research shows that by the fall of 2021, vaccination rates for older adults plateaued at around 80% (7). While the reasons for this gap are complex, experts believe that a hospital-centric healthcare system that inherently reduces resources that could otherwise be allocated for care in alternative settings are ill-equipped to support seniors experiencing mobility and other age-related challenges (8). Vulnerable subpopulations that comprise the wider group of older adults, such as racial/ethnic minorities and socioeconomically disadvantaged communities, are also at disproportionately high risk of disease transmission and burden (9). Consequently, these increased risks may prompt concerns among vulnerable seniors about receiving vaccinations in public settings such as hospitals and pharmacies. Further, vaccination sites may be less easily accessible to high-risk subpopulations overall (10). Altogether, these barriers are likely to prevent those at highest risk for severe illness and death from receiving a vaccine in a timely manner. More broadly, sex, race, and economic disparities in influenza and human papillomavirus vaccination have been observed (11, 12).

In order to mitigate potential inequities in COVID-19 vaccination, key public health and medical stakeholders have called for vaccine distribution efforts to prioritize vulnerable groups. Previously identified solutions include: simplifying the registration process, prioritizing of areas most severely affected by COVID-19 and with high indexes of economic hardship, partnering with local organizations, and assisting those with mobility barriers (13). In-home visits have been proposed as one way to help vaccinate community-dwelling older adults with mobility issues (3, 14). Evaluations of pre-pandemic programs designed to vaccinate seniors against the flu and other viruses in their own homes indicate that such initiatives can be successful in increasing vaccination rates (15–17). Indeed, policymakers have signaled support: in 2021, the Department of Health and Human services issued a brief cataloging concerns around barriers preventing homebound seniors from accessing COVID-19 vaccines (18), and Medicare increased reimbursement for in-home COVID-19 vaccinations (19).

Though literature on the design and execution of COVID-19 in-home vaccination programs is emerging (20, 21), formal evaluations have been scarce, and despite calls to collect vaccine recipients' demographic information in order to ensure that those receiving vaccines match those with the highest COVID burden (13), there is limited data whether in-home vaccination initiatives were successful in reaching those who belong to multiple demographic groups that compound their access barriers and risk profile. The objective of this study was to evaluate whether an in-home COVID-19 vaccination program met its goal of vaccinating high-risk, high-burden populations by reflecting on lessons learned from a program evaluation standpoint and by conducting a formal analysis of the demographic and health-related data collected from vaccine recipients. The latter involved: (1) describing the demographic and health characteristics of adults enrolled in the program, and (2) reporting on motivations for seeking or receiving in-home care as an alternative to traditional vaccination sites, and how motivations might differ by age, sex, and racial/ethnic groups.

## Materials and methods

### Overview of program

During the phased COVID-19 vaccine roll-out period (February to May 2021), Ro, a healthcare technology company based in New York, NY, USA, worked with the New York State Department of Health (NYS DOH) to implement a novel in-home vaccination program for adults aged 65 years and older. Together, Ro and NYS DOH identified Yonkers, a county with a high proportion of seniors and ethnic minorities, as a high priority area for residents to receive vaccinations. Outside of New York City, Yonkers had reported one of the highest COVID-19 caseloads in New York in March 2020 (22). Eligible community members were made aware of the program via community outreach. In order to simplify the enrollment process, age and county of residence were the only inclusion criteria.

### Clinical support and vaccine recipient safety

Prior to scheduling their appointments, vaccine recipients were screened for contraindications and allergies. Throughout the program, vaccine recipients had access to easy-to-understand education materials about COVID-19 vaccine side effects and adverse event (AE) management. For ongoing support, Ro's nurse hotline was available for vaccine recipients to ask questions regarding the vaccine, vaccination process, and potential side effects. NYS-licensed registered nurses and pharmacists were recruited as vaccinators. All vaccinators were required to be fully vaccinated prior to starting in-home visits. Live training sessions and weekly check-ins were instituted to communicate the clinical protocols and processes of the in-home visit. Trainings included modules on AE reporting, technological aspects of care delivery, and administering contingency doses. A dedicated, full-time field operations team provided ongoing, immediate communication with vaccinators in the field. An on-call board-certified physician was available for escalation of AE reporting.

### Appointment scheduling

The program's technological infrastructure was designed so that appointments could be scheduled by eligible individuals or, if digital literacy was a challenge, by their caregivers. To further ensure we reached those with digital access/literacy challenges, Ro and NYS DOH worked with the Yonkers Mayor's Office and YOFA to engage family members, caregivers, and guardians to sign up and coordinate appointments on behalf of eligible vaccine recipients. YOFA managed outreach to other local organizations that were able to identify aging and homebound populations, such as the disabled and the blind. The vaccine drive's software platform was developed to support scheduling, vaccine administration reporting, and collection of demographic information. The team tested foreseeable technological risks before program implementation. Compliance with HIPAA, federal, state, and other regulatory reporting requirements were built into the program's technology. Workpath, a proprietary software platform and subsidiary of Ro designed to provide logistical support for in-home health care, was used to coordinate and dispatch vaccinators to vaccine recipients' homes, and alert them in real-time about their appointments.

### Operations and logistics

NYS DOH provided Ro use of two federal and state approved vaccine storage sites: St. Joseph's Hospital and the Westchester County Center. These centralized locations served as the starting and ending point for vaccinators. Onsite teams at the vaccine storage site coordinated with vaccinators to determine storage, pickup, and drop-off processes in advance. During pickup, vaccinators were equipped with vaccine storage/administration supplies and education materials. Because vaccines were scarce at the time the program was launched, standby potential vaccine recipients were recruited from a centralized location, i.e., apartment complexes and local police/fire stations. This enabled vaccinators to congregate at the end of the day and help each other administer leftover vaccines. Members of the Ro team created and ran machine learning models to optimize travel routes for maximum vaccine efficacy. Contingency planning also included instituting emergency protocols for vaccinators to respond to AEs and reportable home situations, such as domestic and elder abuse. Moderna and Johnson & Johnson (J&J) vaccines were supplied by NYS DOH. Vaccine recipients were given Moderna or J&J depending on availability of the vaccine as supplied by NYS DOH. Moderna vaccines were administered February-May 2021, and J&J vaccines were administered April-May 2021, accounting for the pause from the CDC.

Translation services were used when English was not a vaccine recipient's first or preferred language. Initially, the program used the help of bilingual community members and Google Translate services but this was not ultimately sustainable, as these sources of translation lengthened the duration of visits and were not consistently reliable for the exchange of medical information. This in turn impacted waste mitigation efforts and vaccinator workload. Ultimately, Jeenie, a live web-based app specifically intended for medical translation, was used to translate for non-English speaking vaccine recipients.

### Data collection and analysis

During the home visit, all vaccinators were required to observe vaccine recipients for signs of allergic reactions or adverse events immediately following vaccine administration. During this observation period, vaccinators verbally asked vaccine recipients demographic and health questions from an online questionnaire. Vaccinators were instructed not to read the answer choices aloud, with the exception of COVID-related health conditions. This was in order to prevent bias in vaccine recipients' responses. The online questionnaire also allowed vaccinators to submit vaccine recipient consent forms, to record vaccine information. Vaccinators were also given paper copies of this questionnaire in case of technical difficulties. For data collection integrity, paper, and digital copies were cross-referenced. In the event that a vaccine recipient experienced vaccine-related side effects, vaccinators submitted reports to the Vaccine Adverse Event Reporting System (VAERS).

In total, the program reached 1,076 vaccine recipients. Of these patients, 1,063 received either a J&J dose (*n* = 85) or at least a Moderna first dose (*n* = 978). Those who only received a Moderna second dose (*n* = 13) were ultimately omitted from analyses due to missingness in demographic data. This missingness was due to the structure of intake forms, which prioritized efficiency of vaccine administration over demographic data collection. People who only received one dose of the Moderna vaccine represented either standby cases who received contingency vaccines in order to mitigate waste, or who could not be reached to schedule an additional appointment. People under the age of 65 who received contingency vaccines (*n* = 136) were also omitted from analyses. The final analytical sample consisted of 927 vaccine recipients. Age categories of youngest-old (65–74 years), middle-old (75–84 years), and oldest-old (85 years and older) were selected based on previous studies of older adults (23, 24). Vaccine recipients were able to choose multiple answers for the race/ethnicity question; for a more parsimonious analysis, those who selected more than one answer or who chose “Other” were counted as a separate category. Conditions that were considered COVID-relevant comorbidities were determined using CDC guidelines (6). Several of the answer choices for seeking/receiving in-home vaccinations were grouped together based on similarities in theme. *Convenience and accessibility issues/concerns unrelated to disability* contained the following answer choices: “I couldn't get the vaccine at a traditional site (e.g., line/wait times were too long),” “The closest vaccination site was too far away,” and “In-home vaccination is more convenient.” *Concern, hesitancy, and/or lack of resources about COVID-19 vaccination* contained the following answer choices: “I didn't think I needed the vaccine,” “I didn't know I could get it somewhere other than in my home,” “I didn't know how to get an appointment at a traditional site (e.g., difficulty using online sign-up),” and “I don't have a regular doctor and was concerned I would be able to follow up if things went wrong.” *I am home-bound due to mobility, cognitive, or other disability-related reasons* and *I am avoiding high-risk/public areas due to COVID concerns* were kept as their own categories. The answer choice “My job or other responsibilities prevent me from going to a traditional site during open hours” was excluded due to a small analytical subgroup (*n* = 9).

For each category of number of comorbidities reported, differences in age category were compared. For each reason cited as motivation for receiving and/or seeking in-home care as an alternative to receiving the vaccine at a traditional vaccination site, differences in age, sex, and race/ethnicity were compared. Differences in distribution of race/ethnicity across reasons cited were visualized as a heatmap. Of each reason cited, the darker colors represent a higher percentage of the racial/ethnic group citing that reason. Only Black/African American, Latinx, Asian American/Pacific Islander, and White/Caucasian were included, due to small sample sizes for those who listed other races/ethnicities.

Chi-squared tests were used as statistical tests of difference. A *p*-value of <0.05 was considered significant, and a *p*-value between 0.05 and 0.10 was considered marginally significant. Questions for which vaccine recipients answered “prefer not to answer” or “don't know” were considered missing data. R version 4.0.3 was used.

## Results

### Key program evaluation lessons learned

Several learnings emerged throughout the course of the in-home vaccination program. First, we had not initially accounted for the time needed to accommodate vaccine recipients who expressed vaccine hesitancy. In response, we updated appointment time windows for extended vaccine education during the visit and developed workflows to ensure that vaccinators were continually equipped with up-to-date vaccine education information and guidance from state and federal health organizations. This was especially important in responding to vaccine recipient concerns about the CDC-recommended J&J vaccine pause, which occurred in the middle of the program.

Second, because our vaccine recipient population consisted of homebound seniors, we had not anticipated the need for rescheduling appointments and thus did not design the scheduling system to accommodate this need. Initially, the only way for vaccine recipients to reschedule was to fill out a new online form, creating the issue of duplicate data entries. We ultimately built the capability to submit back-dated reports into our technological infrastructure so that we could cross-reference paper reports to revise missing/inaccurate information in virtual reports. On the day of, close communication with building managers helped the operations team understand whether they could expect vaccine recipients to be available later that day. If they were, vaccine recipients who missed their initial appointment time could opt to receive their vaccination during end-of-day contingency administration. To fill vacancies where possible, the operations and hotline teams rescheduled vaccine recipients with later appointments to be seen earlier. Both vaccine hesitancy and rescheduling challenges might have contributed to the small percentage of vaccine recipients who did not receive their required second dose.

Another challenge and program limitation was digital sign up. Using a digital platform reduced administrative burden on the program side but might have created access barriers for some. To some extent the program design anticipated that some vaccine recipients might be dependent on caregivers or community outreach workers to sign them up and create appointments, but this led to instances where vaccine recipients themselves did not have a phone number or email address where they could be reached to coordinate appointments. We found it effective to work directly with apartment building managers and staff, who helped coordinate on their residents' behalf. Future programs might consider a dedicated telephone hotline might remove access challenges for socially isolated seniors without sufficient internet or computer access, or with limited digital literacy.

### Adverse events

Five hundred and thirty-four patients reported mild side effects, including pain at injection site, redness at injection site, swelling at injection site, nausea/vomiting, fever, muscle aches, tiredness, chills, and headache. Five reports were submitted to the Vaccine Adverse Event Reporting System (VAERS) for patients who experienced a more serious health event some time after receiving a dose, though it was undetermined whether these cases were related to the vaccine.

### Results from analysis on demographic and health-related data

Table 1 shows demographic and health characteristics of the vaccine recipient population. The median age was 79 ± *SD* 9.0 years, and there were approximately the same proportion of vaccine recipients in each age group (32.0% vaccine recipients in the youngest-old age group, 36.6% in the middle-old group, and 31.4% in the oldest-old age group). The oldest vaccine recipient was 107 years old. No age data were missing. Of those vaccine recipients with non-missing data, most vaccine recipients were female (63.1%), identified as part of a racial/ethnic minority (66.4%), reported an annual income of <$25,000 (57.7%), and received a high school degree or less (68.1%). Most vaccine recipients (72.0%) reported having at least one conditions associated with increased risk of severe COVID-19 disease, and 39.2% reporting reported having multiple COVID-related comorbidities. The most commonly reported conditions were high blood pressure (57.3%), heart conditions (22.9%), and/or diabetes (21.3%). Most vaccine recipients learned about the in-home vaccination program through a community organization (59.8%) and/or a friend or family member (22.0%). The majority of vaccine recipients received health insurance through Medicare (74.2%), and almost a quarter received health insurance through Medicaid (21.6%).

**Table 1:** Demographic and health characteristics of patients seeking care from an in-home COVID-19 vaccination program.

**Characteristic**	**Value**
Median age, years (*SD*)	79 (9.0)
Age range, years	65–107
Age category, % with non-missing data (*n*)	100 (927)
Youngest-old (65–74 years), % of those with non-missing age data (*n*)	32.0 (297)
Middle-old (75–84 years), % of those with non-missing age data (*n*)	36.6 (339)
Oldest-old (≥85 years), % of those with non-missing age data (*n*)	31.4 (291)
Sex, % with non-missing data (*n*)	92.1 (854)
Female, % of those with non-missing sex data (*n*)	63.1 (539)
Male, % of those with non-missing sex data (*n*)	36.9 (315)
Race/ethnicity, % with non-missing data (*n*)	83.2 (771)
Black/African American, % of those with non-missing race/ethnicity data (*n*)	11.2 (86)
Native American/American Indian, % of those with non-missing race/ethnicity data (*n*)	0.1 (1)
Latinx, % of those with non-missing race/ethnicity data (*n*)	29.7 (229)
Asian American/Pacific Islander, % of those with non-missing race/ethnicity data (*n*)	23.7 (183)
White/Caucasian, % of those with non-missing race/ethnicity data (*n*)	33.6 (259)
Multiple races listed/other, % of those with non-missing race/ethnicity data (*n*)	1.7 (13)
Household income, % with non-missing data (*n*)	25.2 (234)
<$25,000, % of those with non-missing income data (*n*)	57.7 (135)
$25,000–$49,999, % of those with non-missing income data (*n*)	23.5 (55)
$50,000–$149,999, % of those with non-missing income data (*n*)	15.4 (36)
$150,000–$199,999, % of those with non-missing income data (*n*)	1.3 (3)
$200,000 or more, % of those with non-missing income data (*n*)	2.1 (5)
Educational attainment, % with non-missing data (*n*)	42.9 (398)
Less than high school, % of those with non-missing education data (*n*)	29.4 (117)
High school or GED, % of those with non-missing education data (*n*)	38.7 (154)
Some college or 2 year/vocational/AA degree, % of those with non-missing education data (*n*)	16.8 (67)
4-year degree, % of those with non-missing education data (*n*)	12.1 (48)
Post grad, % of those with non-missing education data (*n*)	3.0 (12)
Number of COVID-relevant comorbidities, % with non-missing data (*n*)	65.5 (607)
No comorbidities, % of those with non-missing comorbidity data (*n*)	28.0 (170)
1 comorbidity, % of those with non-missing comorbidity data (*n*)	32.8 (199)
Multiple (>1) comorbidities, % of those with non-missing comorbidity data (*n*)	39.2 (238)
Type of COVID-relevant comorbidities, % with non-missing data (*n*)*	65.5 (607)
Chronic kidney disease, % of those with non-missing comorbidity data (*n*)	3.1 (19)
Lung disease, % of those with non-missing comorbidity data (*n*)	5.3 (32)
Asthma, % of those with non-missing comorbidity data (*n*)	4.8 (29)
Heart condition, % of those with non-missing comorbidity data (*n*)	22.9 (139)
Overweight/obesity, % of those with non-missing comorbidity data (*n*)	13.3 (81)
High blood pressure, % of those with non-missing comorbidity data (*n*)	57.3 (348)
Diabetes, % of those with non-missing comorbidity data (*n*)	21.3 (129)
Sickle cell disease, % of those with non-missing comorbidity data (*n*)	0.2 (1)
Down syndrome, % of those with non-missing comorbidity data (*n*)	0 (0)
Weakened immune system due to drugs or therapy, % of those with non-missing comorbidity data (*n*)	3.5 (21)
Weakened immune system due to medical condition, % of those with non-missing comorbidity data (*n*)	1.5 (9)
Cancer other than leukemia or lymphoma, % of those with non-missing comorbidity data (*n*)	3.5 (21)
How they heard about the in-home vaccination program, % with non-missing data (*n*)	74.0 (686)
Friend or family, % of those with non-missing referral data (*n*)	22.0 (151)
Pamphlet or written material, % of those with non-missing referral data (*n*)	7.9 (54)
Ro website, % of those with non-missing referral data (*n*)	2.6 (18)
Social media, % of those with non-missing referral data (*n*)	0.7 (5)
Someone from a community organization, % of those with non-missing referral data (*n*)	59.8 (410)
Other, % of those with non-missing referral data (*n*)	12.4 (85)
Health insurance, % with non-missing data (*n*)	67.9 (629)
Medicare, % of those with non-missing insurance data (*n*)	74.2 (467)
Medicaid, % of those with non-missing insurance data (*n*)	21.6 (136)
Private (purchased), % of those with non-missing insurance data (*n*)	7.3 (46)
Private (via employer or family member's employer), % of those with non-missing insurance data (*n*)	4.0 (25)
Uninsured, % of those with non-missing insurance data (*n*)	0.5 (3)
Other, % of those with non-missing insurance data (*n*)	17.2 (108)

Table 2 shows the number of COVID-related comorbidities, by age group. The number of COVID-related comorbidities did not differ significantly by age group (*p* = 0.18).

**Table 2:** Number of COVID-related comorbidities listed, age categories (*n* = 607).

**Number of COVID-related comorbidities**	**All patients,** **% (*n*)**	**Youngest-old** **(65–74 years),** **% (*n*)**	**Middle-old** **(75–84 years),** **% (*n*)**	**Oldest-old** **(≥85 years),** **% (*n*)**	***P*-value**
Patients with non-missing data	100 (607)	32.8 (199)	35.1 (213)	32.1 (195)	0.18
No comorbidities	28.0 (170)	37.1 (63)	29.4 (50)	33.5 (57)	–
1 comorbidity	32.8 (199)	27.1 (54)	39.7 (79)	33.2 (66)	–
Multiple (>1) comorbidities	39.2 (238)	34.5 (82)	35.3 (84)	30.3 (72)	–

Table 3 shows differences in vaccine recipients' motivations for seeking in-home care as an alternative to vaccination at traditional sites, by age group. Among all patients, the most commonly cited motivation was being home-bound due to mobility, cognitive, or other disability-related reasons (44.5%). This was followed by convenience and accessibility issues/concerns unrelated to disability (35.3%); avoiding public areas due to concerns about COVID-19 transmission (27.2%); and concern, hesitancy, and/or lack of resources about COVID-19 vaccination (21.9%). When examining differences between age groups, we found that vaccine recipients who said they sought in-home vaccination because they were home-bound due to disability were most likely to be aged 85 years or older; 38.9% of those citing this reason were in the oldest-old age group (*p* = 0.004). Vaccine recipients who cited convenience and accessibility issues unrelated to disability were more likely to be in the middle-old age group (38.9%), though this difference was marginally significant (*p* = 0.05). There were no differences in age group between vaccine recipients who cited avoidance of public areas due to concerns about COVID transmission or concern, hesitancy, and/or lack of resources about COVID-19 vaccination (*p* > 0.10).

**Table 3:** Motivations for seeking in-home care as an alternative to traditional vaccination sites, age categories (*n* = 677).

Motivation^*^	**All patients,** **% (*n*)**	**Youngest-old** **(65–74 years),** **% citing this reason** **(*n*)**	**Middle-old** **(75–84 years),** **% citing this reason** **(*n*)**	**Oldest-old** **(≥85 years),** **% citing this reason** **(*n*)**	***P*-value**
Patients with non-missing data	100 (677)	32.3 (219)	35.5 (240)	32.2 (218)	–
Home-bound due to mobility, cognitive, or other disability-related reasons	44.5 (301)	28.9 (86)	32.6 (98)	38.9 (117)	0.004
Convenience and accessibility issues/concerns unrelated to disability	35.3 (239)	34.7 (83)	38.9 (93)	26.4 (63)	0.05
Avoiding public areas due to COVID concerns	27.2 (184)	30.4 (56)	34.8 (64)	34.8 (64)	0.66
Concern, hesitancy, and/or lack of resources about COVID-19 vaccination	21.9 (148)	37.2 (55)	33.1 (49)	29.7 (44)	0.37

* Reflects overlapping answers, as patients were able to select more than one reason.

Table 4 shows differences in motivations for seeking in-home care, by sex. Compared to males, females were more likely to report being home-bound as a reason for seeking/receiving in-home vaccination (67.8%, *p* = 0.047). There was a noticeably smaller proportion of females who reported a reason related to a concern, hesitancy, and/or lack of resources about COVID-19 vaccination (56.6%); this was marginally significant (*p* = 0.08). We did not observe any sex differences in those who reported seeking in-home care due to avoidance of public areas or convenience/accessibility issues unrelated to disability were observed (*p* > 0.10).

**Table 4:** Motivations for seeking in-home care as an alternative to traditional vaccination sites, sex (*n* = 646).

Motivation^*^	**All patients, % (*n*)**	**Females, % citing this reason (*n*)**	***P*-value**
Patients with non-missing data	100 (646)	63.3 (409)	–
Home-bound due to mobility, cognitive, or other disability-related reasons	44.1 (285)	67.8 (193)	0.047
Convenience and accessibility issues/concerns unrelated to disability	35.8 (231)	64.1 (148)	0.83
Avoiding public areas due to COVID concerns	26.8 (173)	67.1 (116)	0.27
Concern, hesitancy, and/or lack of resources about COVID-19 vaccination	22.1 (143)	56.6 (81)	0.08

* Reflects overlapping answers, as patients were able to select more than one reason.

**Figure 1:**
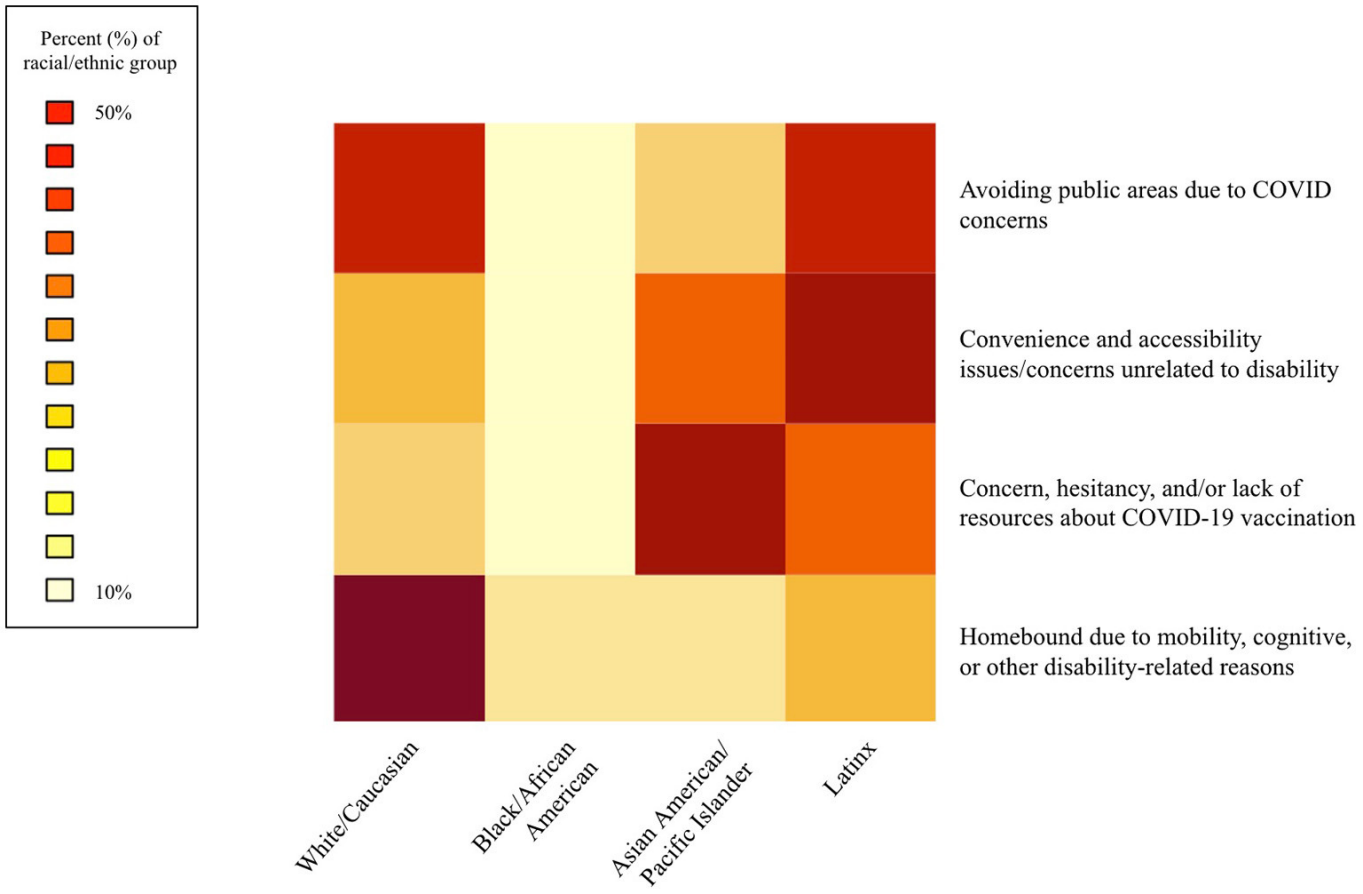
Motivations for seeking in-home care as an alternative to traditional vaccination sites, race/ethnicity (*n* = 670).

Figure 1 shows motivations for seeking in-home care as an alternative to traditional vaccination sites, by race/ethnicity. There were significant differences in race/ethnicity for all motivations (*p* < 0.05), except for avoidance of public areas (*p* = 0.42); however, proportionally more Black/African American (13.3%) and Latinx (33.1%) vaccine recipients reported avoiding public areas than other races. White/Caucasian (52.8%) and Black/African American vaccine recipients were more likely than other races to report being home-bound due to disability (13.2%, *p* < 0.0001). Those citing convenience and accessibility issues unrelated to disability were more likely to identify as a race/ethnicity other than White (*p* = 0.003). Vaccine recipients who identified as Asian American/Pacific Islander were more likely to seek/receive in-home care due to concern, hesitancy, and/or lack of resources about COVID-19 vaccination (41.4%, *p* < 0.0001).

## Discussion

This in-home COVID-19 vaccination program aimed to serve vulnerable populations most at risk for severe COVID-19 disease; the objective of this study was to determine whether the program achieved its aim, both by reflecting on lessons learned from implementation and by conducting a formal analysis of demographic and health-related data. In total, 1,063 people were fully vaccinated. While we consider this to be an indicator of success, one limitation of this evaluation is the lack of cost/benefit analysis due to the nature of funding for the program. This is a highly useful metric for other organizations considering launching a similar initiative. However, from a process evaluation standpoint, we learned several operational lessons that can inform future efforts how to maximize program efficiency and reduce administrative costs in future iterations of in-home care delivery programs. This includes accounting for the amount of provider-facing time that patients may need to ask questions about the care they are receiving, as well as surveying the population in order to gain a clear and thorough understanding of their specific needs. The latter is essential to informing what additional services or resources may be needed; in our program we found that, in order for the ultimate goal of vaccine delivery to actually be effective, we needed comprehensive scheduling capabilities, language translation services, and phone alternatives to digital sign-ups.

The main limitation of the outcome evaluation was missing data, which may have biased reporting. For example, White vaccine recipients may have seemed more likely to cite being homebound as a motivation for seeking in-home vaccination because White vaccine recipients were more likely to answer questions. However, as minority populations may be less likely to respond to race/ethnicity survey questions (25), there is a possibility that members of vulnerable populations made up an even larger portion of the vaccine recipient population than observed in the existing data. To alleviate discomfort in disclosing sensitive information, future iterations of this program should prioritize written collection of demographic data, as data missingness was at least partially attributable to the verbal delivery of sensitive questions. Thoughtful collection of these types of data is integral to tracking program progress in equitable distribution of care. Other potential limitations included lack of generalizability due to the specificity of the study population (older adults living in Yonkers, NY), and inaccuracy of income data collected from retirement-age individuals. While the latter is a limitation inherent to this population, it may be inferred that this population's concerns with vaccination (especially in traditional, in-person settings) are consistent with other populations with similar demographic characteristics (older adults with additional indices of social vulnerability). This is especially important when considering disease prevention in older adults amidst the continued emergence of new COVID-19 variants.

Limitations notwithstanding, this innovative in-home COVID-19 vaccination program allowed medical professionals to vaccinate high-risk seniors despite mobility, accessibility, transmission risk, and vaccine hesitancy barriers. Data collection was a focal point of the program and executed via (1) a simplified registration process and (2) the capture of key demographic and health information, in order to assess whether the program met its goals of prioritizing areas with the highest COVID burden, communities with high indexes of economic hardship, and people with mobility and access barriers. These were in line with expert recommendations (13).

Indeed, of those who answered demographic questions, most were part of a racial/ethnic minority group and reported lower household income and educational attainment. The majority of vaccine recipients were women. Though early research indicated that, compared to men, women were more likely to express hesitancy about COVID-19 vaccination, these studies were conducted prior to the availability of vaccines and asked about hypothetical behavior (26, 27). Once vaccines were available in the US, women were more likely to seek and receive COVID-19 vaccinations (28). Older women appear more likely to utilize in-home health services, mainly attributable to women's greater health needs (29). More research is needed to understand the drivers behind lower uptake for men and whether future programs can mitigate this through targeted outreach.

The social vulnerability of this vaccine recipient population was underscored by the high proportion of people who reported receiving health insurance through Medicare and Medicaid. Most reported having at least one COVID-related health condition, regardless of age. The most commonly reported conditions were high blood pressure, heart conditions, and diabetes. Of note, the majority of vaccine recipients reported that they heard about the in-home vaccination program through a community organization, emphasizing the importance of partnership with local and government organizations. When asked why they sought or received in-home COVID-19 vaccination, those who cited being home-bound due to disability were more likely to be older, female, and White/Caucasian or Black/African American. This was also the most commonly cited reason overall, meaning the program achieved its original goal of reaching homebound seniors, particularly the oldest subset of the 65+ population. Those citing convenience and accessibility issues unrelated to disability were more likely to be in the middle range of older adulthood and to be non-White. This may reflect well-documented environmental and transportation barriers to healthcare for minority populations (30). Though it was not statistically significant, proportionally more Black and Latinx vaccine recipients reported seeking in-home care in order to avoid public areas. Black and Latinx populations are known to be disproportionately affected by COVID, due to greater vulnerability to risk factors that increase risk of transmission (i.e. fewer economic opportunities to work in non-public facing jobs) and health inequities that promote the development of COVID-related comorbidities (9). Those citing a reason related to concern, hesitancy, and/or lack of resources about vaccination were more likely to be Asian American/Pacific Islander. These may be related to language barriers and insulation from knowledge of social services often experienced by older AAPI adults (31). Variation in responses as to why seniors opted for in-home vaccination underscore the importance of one of the program's guiding principles: attempting to define a measure of home-boundedness for program inclusion criteria, e.g., a documented disability, would have resulted in the exclusion of many community members from the program. Those exclusions would likely have included a disproportionate amount of non-White recipients. The added administrative burden would have also undermined program administrators' capacity to address logistical challenges, such as ensuring second dose appointments occurred and preventing vaccine waste.

Together, both our program evaluation learnings and our analysis of the population served indicate that our in-home care delivery program was successful in its implementation and can inform future iterations of similar programs. The population receiving in-home vaccination services through this program were mostly non-White, were part of socioeconomically disadvantaged communities, and reported one or more COVID-related health conditions, indicating that the in-home vaccination program was successful in reaching high-need, high-risk seniors. The importance and timeliness of in-home vaccination programs—especially given ongoing concerns and barriers around receiving vaccinations in public areas for those most vulnerable to severe disease—should be considered in light of new COVID-19 variants.

## Data availability statement

The datasets presented in this article are not readily available because disaggregated data containing details about age, sex, race, and other demographics, in combination with the location-specific nature of the study, may become identifiable. Requests to access the datasets should be directed to megan.zhou@ro.co.

## Ethics statement

The studies involving human participants were reviewed and approved by Biomedical Research Alliance of New York Institutional Review Board (BRANY IRB). Written informed consent for participation was not required for this study in accordance with the national legislation and the institutional requirements.

## Author contributions

MSZ was primarily responsible for manuscript writing and for the design and execution of the analytical plan. MB oversaw and coordinated all clinical aspects of the program. JJ and MD oversaw and coordinated logistical aspects of the program, including partnering with NYS DOH. KC and MD documented learnings from the program and assisted in incorporating those learnings into this manuscript. DK, CA, and KQ executed logistical and clinical aspects of the program. TC and JS designed the scheduling and data collection instruments. JK oversaw the design process of the scheduling and data collection instruments. JJ oversaw logistics and communication of patient population information with NYS DOH. JJ and JK coordinated between all organizations involved in the in-home vaccination program. KS was responsible for ensuring data integrity and designing data collection structure. LB oversaw and provided input on all aspects of manuscript writing and the final analytical plan. All authors contributed to manuscript writing and approved the final manuscript before its submission.

## Conflict of interest

At the time this program was conducted, all authors were full-time employees of and had stock options in Ro, the telehealth company that provided the data for this study.

## Publisher's note

All claims expressed in this article are solely those of the authors and do not necessarily represent those of their affiliated organizations, or those of the publisher, the editors and the reviewers. Any product that may be evaluated in this article, or claim that may be made by its manufacturer, is not guaranteed or endorsed by the publisher.
